# THE SOCIAL, ECONOMIC, AND MORTALITY CONSEQUENCES OF THE FAILURE OF THE FREEDMAN’S SAVINGS BANK

**DOI:** 10.1093/geroni/igae098.1751

**Published:** 2024-12-31

**Authors:** John Warren, Jonas Helgertz, Michael Esposito, Elizabeth Wrigley-Field, J David Hacker

**Affiliations:** University of Minnesota, Minneapolis, Minnesota, United States; Lund University, Lund, Skane Lan, Sweden; University of Minnesota, Minneapolis, Minnesota, United States; University of Minnesota, Minneapolis, Minnesota, United States; University of Minnesota, Minneapolis, Minnesota, United States

## Abstract

The Freedman’s Savings Bank was chartered in 1865 to serve newly emancipated people across 17 states. Driven by mismanagement and fraud by the bank’s white officials, the bank failed in 1874 ─ costing many depositors large savings. Although there is debate about the short-term financial benefits for Black people of the bank’s creation, there has been no research on the longer-term effects of the bank’s failure on social, economic, or mortality outcomes among depositors who lost their savings. Using linked records from the complete-count 1860 through 1940 U.S. Censuses and exceptionally detailed records on the bank’s branches, deposits, and losses, we use a variety of empirical estimation strategies to estimate the effects of the bank’s failure on the life course outcomes of Black depositors and their children. This work speaks more broadly to the effects of negative economic shocks on the long-term well-being of older people.

